# Superconductivity in Ti_4_O_7_ and *γ*-Ti_3_O_5_ films

**DOI:** 10.1038/s41598-017-12815-4

**Published:** 2017-10-02

**Authors:** K. Yoshimatsu, O. Sakata, A. Ohtomo

**Affiliations:** 1 Department of Chemical Science and Engineering, Tokyo Institute of Technology, 2-12-1 Ookayama, Meguro-ku, Tokyo, 152-8552 Japan; 2 Synchrotron X-ray Station at SPring-8, National Institute for Materials Science (NIMS), 1-1-1 Koto, Sayo-cho, Sayo-gun, Hyogo, 679-5148 Japan; 3 Materials Research Center for Element Strategy (MCES), Tokyo Institute of Technology, 4259 Nagatsuta-cho, Midori-ku, Yokohama, 226-8503 Japan

## Abstract

Titanium dioxide is one of the most popular compounds among simple oxides. Except for the fully oxidized titanate, titanium oxides have partially filled *d* states and their exotic properties have captured attention. Here, we report on the discovery of superconductivity in Ti_4_O_7_ and *γ-*Ti_3_O_5_ in a thin film form. The epitaxial Ti_4_O_7_ and *γ-*Ti_3_O_5_ thin films were grown using pulsed-laser deposition on (LaAlO_3_)_0.3_–(SrAl_0.5_Ta_0.5_O_3_)_0.7_ and *α*-Al_2_O_3_ substrates, respectively. The highest superconducting transition temperatures are 3.0 K and 7.1 K for Ti_4_O_7_ and *γ-*Ti_3_O_5_, respectively. The mechanism behind the superconductivity is discussed on the basis of electrical measurements and previous theoretical predictions. We conclude that the superconductivity arises from unstabilized bipolaronic insulating states with the assistance of oxygen non-stoichiometry and epitaxial stabilization.

## Introduction

**Figure 1:**
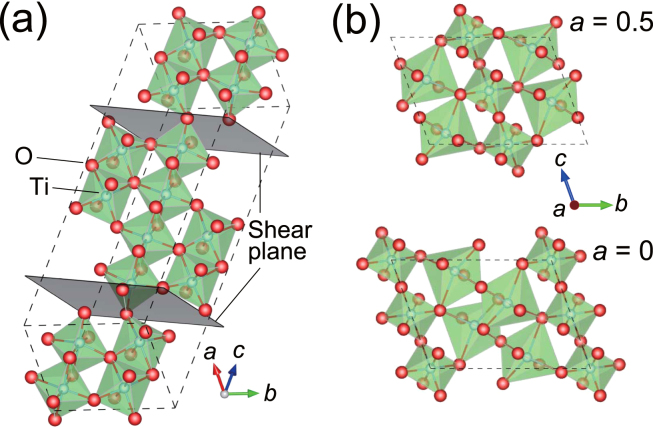
Crystal structures of titanates. Schematics of the crystal structures for (**a**) the first member of Magnéli-phase Ti_4_O_7_ and (**b**) *γ*-Ti_3_O_5_.

In the periodic table, titanates are the first group of simple oxides, which are defined as oxides consisted of a kind of the cation and oxygen ion(s), indicating metallicity, and all the simple oxides of scandium or much lighter elements are insulating. Therefore, the choice of titanates is favourable for large electron–phonon coupling. Figure 1(a) shows a schematic of the crystal structure for Ti_4_O_7_. Ti_4_O_7_ is the first member of Magnéli phase [a triclinic cell (*a* = 5.597 Å, *b* = 7.125 Å, *c* = 20.429 Å, *α* = 67.7°, *β* = 57.16°, *γ* = 108.76°)]^1,2^ that exhibits unique low-dimensional structures characterized by shear planes. These shear planes correspond to the rutile TiO_2_ (121) planes and amputate the edge-shared infinite TiO_6_ chains at every *n* TiO_6_ blocks with shifting by a half of the unit cell. In the nominal composition, a TiO_6_ tetramer has two electrons occupying the Ti 3*d* states. Trititanium pentoxide (Ti_3_O_5_) with polymorphisms (*α*-, *β*−, *γ*−, *δ*−, and *λ*-phases) is a neighbour of the Magnéli phase^3–7^. *γ*-Ti_3_O_5_ is one of them with a monoclinic cell (*a* = 5.0747 Å, *b* = 9.9701 Å, *c* = 7.1810 Å, *α* = 109.865°)^4^. In contrast to the Magnéli phase, there are no shear planes, as illustrated in Fig. 1(b). However, since the chemical formula is consistent with that of the Magnéli phase (Ti_*n*_O_2*n*-1_ at *n* = 3), it is sometimes designated as the first member of the Magnéli phase. Because of difficulty in the growth of a single crystal due to polymorphism, their physical properties are still under debate. Several studies have dealt with the structural phase transitions accompanying MIT, which are induced under the specific conditions (*α* ↔ *β* at 450 K^3^, *δ* ↔ *γ* at 240 K^4–7^, and *β* ↔ *λ* by irradiation using visible-light pulses^6^).

We find that Ti_4_O_7_ and *γ*-Ti_3_O_5_ films synthesized using epitaxial growth are superconductors with *T*_C_s 3.0 K and 7.1 K, respectively. The temperature dependence of resistivity strongly depended on the growth atmosphere. The Ti_4_O_7_ film grown under a more oxidation condition of oxygen atmosphere exhibited metal–insulator transition (MIT) accompanied by clear hysteresis at ~150 K. The insulating phase was suppressed in the films grown under a less oxidative condition of Ar atmosphere, and the superconducting phase appeared at low temperatures. These results and the previous theoretical prediction suggest that epitaxial stabilization and oxygen non-stoichiometry play key roles in the realization of superconductivity in these titanates.

## Results

### Structural characterization

**Figure 2:**
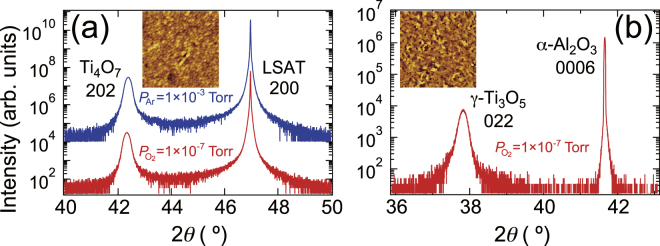
Structural characterization of titanate films. (**a**) Out-of-plane XRD patterns for Ti_4_O_7_ films grown on LSAT (100) substrates under Ar gas at 1 × 10^−3^ Torr (top) and under oxygen gas at 1 × 10^−7^ Torr (bottom). (**b**) Out-of-plane XRD pattern for the *γ*-Ti_3_O_5_ film grown on *α*-Al_2_O_3_ (0001) substrates under oxygen gas at 1 × 10^−7^ Torr. The insets show AFM images (5 µm × 5 µm) taken for the same films. Colour codes are 13 nm and 8 nm in height for (**a**) and (**b**), respectively.

The formation of the Ti_4_O_7_ and γ-Ti_3_O_5_ phases was verified using x-ray diffraction (XRD). The out-of-plane XRD patterns showed intense reflections from the Ti_4_O_7_ films grown on (LaAlO_3_)_0.3_–(SrAl_0.5_Ta_0.5_O_3_)_0.7﻿_(LSAT) (100) substrates and the *γ-*Ti_3_O_5_ film grown on *α*-Al_2_O_3_ (0001) substrates [Fig. 2(a) and (b), respectively]. These substrates are insulating, non-magnetic, and exhibit high reduction resistance, providing advantages in the growth and search of a superconducting sample. Irrespective of the growth condition, Ti_4_O_7_ 202 reflection was detected at 2*θ* = 42.38°, corresponding to *d*_202_ = 2.13 Å. No other film reflections except for the 404 reflection at 2*θ* = 92.60° was detected in wide-range XRD patterns. The *γ*-Ti_3_O_5_ 022 reflection was detected at 2*θ = *37.83°, corresponding to *d*_022_ = 2.38 Å. The out-of-plane single orientation was verified using wide-range XRD patterns (not shown). Surface morphology of the films are shown in the inset of Fig. 2. The small grains were observed and the root mean square roughness was about 1 nm for both films. Their surface morphology was different from that of TiO and Ti_2_O_3_ (see Fig. S1 in Supplementary information)^8^.

Because of various polymorphisms with different ratios of oxygen to titanium, their crystal structures must be carefully distinguished. Then, we used the tilt angle *χ*-dependence of 2*θ*-*θ* XRD profiles to survey the asymmetric film reflections (see Figs S2 and S6 in Supplementary Information). Reflections coming from the substrate and film were found at characteristic *χ* angles. Since the intensities of the film reflections were too weak to determine the *d* values of interplanar spacing precisely, synchrotron radiation XRD measurements were also performed (see Figs S3–S5,S7 and S8 in Supplementary Information). From the *d* values and *χ* angles, we identified the Miller indices as those listed in Tables S2 and S3. In comparison to the previous structural analyses of titanates^1–7^, we concluded that the films grown on LSAT (100) and *α*-Al_2_O_3_ (0001) substrates were Ti_4_O_7_ and *γ*-Ti_3_O_5_, respectively. Furthermore, using the *d* values and Miller indices (Tables S1 and S3), we evaluated lattice parameters of our titanate films: Ti_4_O_7_ film grown under *P*_O2_ = 1 × 10^−7^ Torr (*a* = 5.52 Å, *b* = 7.12 Å, *c* = 20.43 Å, *α* = 67.5°, *β* = 57.3°, *γ* = 108.8°), Ti_4_O_7_ film grown under *P*_Ar_ = 1 × 10^−7^ Torr (*a* = 5.52 Å, *b* = 7.11 Å, *c* = 20.46 Å, *α* = 67.5°, *β* = 57.2°, *γ* = 108.8°), and *γ*-Ti_3_O_5_ film (*a* = 4.99 Å, *b* = 9.80 Å, *c* = 7.06 Å, *α* = 110.3°). The *a*-axis lattice constant of both Ti_4_O_7_ films is smaller than that of bulk. In contrast, *b*- and *c*-axes lattice constants of the former Ti_4_O_7_ films were in agreement with those of bulk. The *b*- (*c*-) axis lattice constant of the latter Ti_4_O_7_ film was smaller (larger) than that of bulk. We note that the *c*-axis length directly corresponds to the Ti–Ti bond length in the TiO_6_ tetramer [see Fig. 1(a)] and *c*-axis lattice constant of the former Ti_4_O_7_ film is larger than that of the latter Ti_4_O_7_ film. For the *γ*-Ti_3_O_5_ film, all of the lattice constants were smaller than those of bulk. The lattice parameters of the titanate films and bulk are listed in Tables S2 and S4 for comparison.

Formation of the different titanate phases under the identical growth condition suggests that epitaxial effects play an important role for stabilizing the Ti_4_O_7_ and *γ*-Ti_3_O_5_ films on each substrate (see Fig. S9 in Supplementary Information). In fact, we have grown neither *γ*-Ti_3_O_5_ films on LSAT (100) substrates nor Ti_4_O_7_ films on *α*-Al_2_O_3_ (0001) substrates. The in-plane epitaxial relationship between the substrates and films were also investigated and described in Supplementary Information.

### Temperature dependence of resistivity

**Figure 3:**
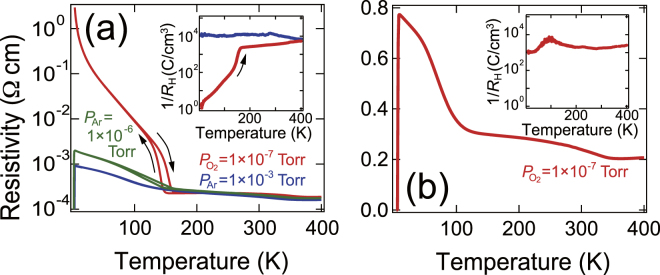
Temperature dependence of resistivity of titanate films. (**a**) Temperature dependence of resistivity for Ti_4_O_7_ films grown under three different conditions. The inset shows the temperature dependence of the Hall measurement. (**b**) Temperature dependence of resistivity for the *γ*-Ti_3_O_5_ film. The inset shows the temperature dependence of the Hall measurement.

The electrical properties of the films were investigated using the temperature dependence of resistivity (Fig. 3). The resistivity curves strongly depended on the growth atmosphere for Ti_4_O_7_ films [Fig. 3(a)]. For the film grown under *P*_O2_ = 1 × 10^−7^ Torr, MIT accompanied by clear hysteresis was found at around 150 K, which is in agreement with the behaviour of a bipolaron insulator of bulk Ti_4_O_7_^9–11^. In contrast, the insulating behaviours were strongly suppressed for the film grown under *P*_Ar_ = 1 × 10^−3^ Torr; the upturn in resistivity was weak. The different behaviour across MIT was in agreement with the difference in *c*-axis lattice constants of the Ti_4_O_7_ films: the larger *c*-axis length weakened the Ti^3+^–Ti^3+^ bond in the TiO_6_ tetramers for the Ti_4_O_7_ films grown under *P*_Ar_ = 1 × 10^−3^ Torr. The weak resistivity upturn was also reported on V-doped bulk Ti_4_O_7_^12^. When V content exceeds 0.35 at%, the disordered bipolarons dominate the electronic properties in the insulating phase. If we account for the lower degree of oxidation at *P*_Ar_ = 1 × 10^−3^ Torr, oxygen deficiency would play a similar role to substitution of the Ti site with V and be responsible for the suppression of the insulating states. Furthermore, superconductivity was observed at low temperatures. The Ti_4_O_7_ film grown under an intermediate condition (*P*_Ar_ = 1 × 10^−6^ Torr) exhibited both hysteresis and superconducting characteristics in the resistivity curve (also see Fig. S10 in Supplementary Information). We will refer to the Ti_4_O_7_ films grown under *P*_O2_ = 1 × 10^−7^ Torr (*P*_Ar_ = 1 × 10^−3^ Torr) as insulating (superconducting) ones in the following discussion.

The variation in the Hall coefficient (*R*_H_) during warming exhibited a tendency similar to that of resistivity. At 300 K (10 K), the inverse *R*_H_ was 3.6 × 10^3^ (1.5) and 1.2 × 10^4^ (1.2 × 10^4^) C/cm^3^ for the films grown under *P*_O2_ = 1 × 10^−7^ Torr and *P*_Ar_ = 1 × 10^−3^ Torr, respectively. For the insulating Ti_4_O_7_ film, the temperature dependence of the inverse *R*_H_ [inset of Fig. 3(a)] suddenly decreased at around 150 K, suggesting that the MIT was induced by the depletion of hole carriers. The inverse *R*_H_ at 10 K was four orders of magnitude smaller than that at 300 K. The MIT in the bulk is associated with the formation of bipolarons^9–11^, which remains robust in the insulating Ti_4_O_7_ film at low temperatures. In contrast, the inverse *R*_H_ for the superconducting Ti_4_O_7_ film was almost independent of temperatures, and even the value at 10 K was comparable to that at 300 K, suggesting the suppression of a bipolaronic insulating state.

The temperature dependence of the resistivity for the *γ*-Ti_3_O_5_ film exhibited a complex curve along three electronic phase transitions: MIT around 350 K, insulator–insulator transition around 100 K, and superconducting transition [Fig. 3(b)]. The intermediate transition would be related to the MIT of Ti_4_O_7_ due to their similar transition temperatures. Nevertheless, the resistivity upturn was much weaker, suggesting the suppression of the insulating states, as with the case of the superconducting Ti_4_O_7_ film. The inverse *R*_H_ almost [inset of Fig. 3(b)] remained the same (~10^3^ cm^3^/C) over the entire temperature range. The sign and magnitude of the *R*_H_ also reflected this correspondence.

### Superconducting properties

**Figure 4:**
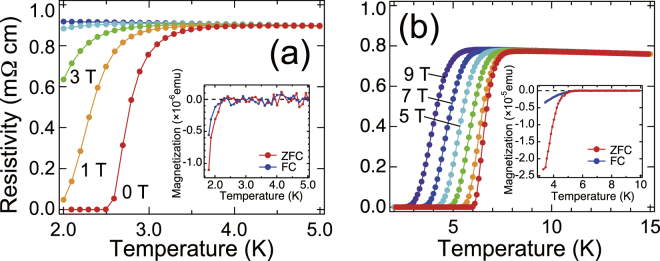
Superconducting properties of titanate films. (**a**) Temperature dependence of resistivity of the Ti_4_O_7_ film grown under *P*_Ar_ = 1 × 10^−3^ Torr at low temperatures. (**b**) Temperature dependence of resistivity of the *γ*-Ti_3_O_5_ film at low temperatures. The insets of (**a**) and (**b**) show temperature dependence of magnetization for (**a**) the superconducting Ti_4_O_7_ and (**b**) *γ*-Ti_3_O_5_ films at low temperatures, respectively. FC and ZFC denote field-cooling and zero-field cooling curves, respectively.

The temperature dependence of resistivity around the temperature of liquid helium indicates further similarity between the superconducting Ti_4_O_7_ and *γ*-Ti_3_O_5_ films [Fig. 4(a) and (b), respectively]. The *T*_C_ of Ti_4_O_7_ and *γ*-Ti_3_O_5_ were 3.0 K and 7.1 K for *T*_C,onset_, 2.7 K and 6.6 K for *T*_C,mid_, and 5.8 K and 2.5 K for *T*_C,zero_, respectively. Note that the *T*_C_ of both films exceeded that of other simple-oxide superconductors in bulk [TiO (*T*_C_ = 2.3 K), NbO (*T*_C_~1.4 K), and SnO (*T*_C_ = 1.4 K under 9.3 GPa)]^13–16^. We also note that enhancement of *T*_C_ = ~7 K in TiO films has been reported in recent^17^. The superconducting states were gradually degraded under applied magnetic fields. Here, the magnetic fields were applied perpendicular to the film surface. *T*_C_ shifted toward a lower temperature under a higher magnetic field, and the superconducting phase finally disappeared for the Ti_4_O_7_ film at above 2 K. As for the *γ*-Ti_3_O_5_ films, superconductivity remained robust even under 9 T. In addition, from the temperature dependence of magnetization measurements, where magnetic field was applied parallel to the film surface, clear diamagnetic signals were observed [insets of Fig. 4(a) and (b)], respectively. The observation of diamagnetic signals in field-cooling curves indicates the Meissner effect of bulk superconductivity inTi_4_O_7_ and *γ*-Ti_3_O_5_ films, and roles out major influences arising from impurity, filament, and/or surface states.

## Discussion

Chakraverty *et al*. proposed a theory to predict Superconductivity in Ti_4_O_7_ with the largest *λ*_ep_ value^18–20^. Therefore, experimental verifications for superconductivity in bulk Ti_4_O_7_ were attempted by applying high pressures. However, no superconducting transition was observed under a hydrostatic pressure of up to 5.0 GPa, although the high-temperature metallic phase was extended down to 3 K^9,10^. Our first observation of superconductivity in a Ti_4_O_7_ film demonstrates the importance of the epitaxial thin film. Titanium-based simple oxides with various chemical formulae and polymorphisms easily transform from one to another, and subtle tuning of oxygen stoichiometry causes modulation of carrier density. Epitaxial growth on LSAT substrates enables us to stabilize the Magnéli phase. In fact, the *γ*-Ti_3_O_5_ and Ti_4_O_7_ films can also be grown on different substrates under the same growth conditions (*T*_g_ = 900 °C and *P*_O2_ = 1 × 10^−7^ Torr) (see Fig. S9 in Supplementary Information). The lack of these advantages would be inevitable for hidden superconducting phases in bulk specimens. The MIT of the stoichiometric Ti_4_O_7_ bulk is premised on the bipolaronic interaction^9–11^. Sharp increase in resistivity and hysteresis at the MIT are strong evidence for the bipolaron formation^9–11^. The insulating Ti_4_O_7_ film exhibiting such characteristics can be regarded as a bipolaronic insulator at low temperatures. For a bipolaronic system, the bipolaron density is a key parameter in the electronic phase diagram^19^. Our growth of Ti_4_O_7_ films under Ar atmosphere aims at inducing extra Ti 3*d* electrons by introducing oxygen vacancies which dilute the bipolaron density, resulting in the suppression of the insulating states. In fact, the inverse *R*_H_ of the superconducting film suggests suppression of the bipolaron formation [Fig. 3(a)]. Ti_4_O_7_ films grown on MgAl_2_O_4_ (100) substrates also exhibited superconductivity (see Figs S11 and S12 in Supplementary Information). Thus, the observed superconductivity is intrinsic to the Ti_4_O_7_ phase. Furthermore, superconductors composed of Mg, Al, Ti, and O with *T*_C_ of more than 3 K are not yet known, indicating that any elements from the substrates cannot induce the superconductivity in our samples.

For bulk *γ*-Ti_3_O_5_, the MIT occurs with the structural phase transition at ~240 K^7^. There was no sign of such a structural phase transition at the temperature in the resistivity curve of the *γ*-Ti_3_O_5_ film [Fig. 3(b)], suggesting that the metallic *γ*-phase was stabilized in an epitaxial thin film. The first-principle calculations revealed a one-dimensional conducting pathway along the *c*-axis arising from the density of states at the Fermi level^7^. The low-dimensional electronic structure would lead to the pairing of electrons at ~100 K where MIT occurred in *γ*-Ti_3_O_5_. On the other hand, the small number of studies on *γ*-Ti_3_O_5_ makes it difficult to discuss the strength of the electron–phonon interaction, the formation of bipolarons, and the density of states at the Fermi level. Further investigation will be necessary to reveal the origin of superconductivity as well as several electronic phase transitions.

In summary, we study new superconductors produced from Ti_4_O_7_ and γ-Ti_3_O_5_ films whose *T*_C_ are 3.0 and 7.1 K, respectively. The latter is one of the highest known values among simple oxides. Our investigations on the electronic properties and the previous theoretical prediction suggest that epitaxial stabilization and oxygen non-stoichiometry play key roles in the realization of superconductivity in the titanates.

## Methods

### Thin-Film Preparation

A TiO_x_ ceramic tablet was prepared using a conventional solid-state reaction method. Ti (3 N) and TiO_2_ (4 N) powders with a molar ratio of 1:3 were mixed and pressed into a pellet. This was sintered at 1000 °C for 12 *h* in vacuum. Prior to the film growth, LSAT and *α*-Al_2_O_3_ substrates were annealed in air to obtain a step-and-terrace surface. The annealing conditions were 1200 °C for 3 *h* for (LaAlO_3_)_0.3_–(SrAl_0.5_Ta_0.5_O_3_)_0.7_ (LSAT), and 1100 °C for 3 *h* for *α*-Al_2_O_3_. The films were grown using PLD in an ultra-high-vacuum chamber. KrF excimer laser pulses (5 Hz, 2.0 J/cm^2^) were focused on the TiO_x_ ceramics tablets. The growth temperature was set at 900 °C. The chamber pressure was controlled with the continuous flow of oxygen or Ar gas (6 N purity for both). Ar atoms in the chamber tend to scatter with the lighter oxygen, especially when mean free path of the gaseous species exceeds the target-substrate distance^21,22^. Therefore, introduction of Ar (oxygen) gas during the growth corresponds to reduction (oxidation) of the films. In fact, we have also grown TiO and Ti_2_O_3_ films using PLD in Ar atmosphere (see Fig. S1 in Supplementary Information)^8^. After the growth, the gas flow was stopped immediately, and the samples were quenched to room temperature.

### Characterization of the thin films

Thickness of all the films was ~120 nm, as measured by a stylus profiler. The crystal structures of the films were characterized using XRD with Cu K*α*_1_ radiation (Rigaku, SmartLab) and synchrotron radiation at BL15XU in SPring-8. The photon energy of the synchrotron radiation was set at 15 keV (*λ* = 0.826 Å). The temperature dependence of resistivity was measured using a standard four-probe method with a physical properties measurement system (Quantum Design, PPMS). The temperature dependence of the Hall measurements was also measured using PPMS in a standard six-terminal geometry. The temperature dependence of magnetization was measured using magnetic properties measurement system (Quantum Design, MPMS).

## Electronic supplementary material

Supplemental Information
