# Supplementary material for: Superconductivity in Ti4O7 and γ-Ti3O5 films
Source: Sci Rep. 2017 Oct 2;7:12544. doi: 10.1038/s41598-017-12815-4 (PMC5624959; doi:10.1038/s41598-017-12815-4)
Supplement: Supplementary file 1 — Supplemental Information [file 41598_2017_12815_MOESM1_ESM.pdf]

Supplementary Information

Superconductivity in Ti_4O_7 and γ-Ti_3O_5 films

K. Yoshimatsu[1,*], O. Sakata[2,3], and A. Ohtomo[1,3,*]

[1]*Department of Chemical Science and Engineering, Tokyo Institute of Technology, 2-12-1 Ookayama, Meguro-ku, Tokyo 152-8552, Japan*

[2]*Synchrotron X-ray Station at SPring-8, National Institute for Materials Science (NIMS), Sayo, Hyogo 679-5148, Japan*

[3]*Materials Research Centre for Element Strategy (MCES), Tokyo Institute of Technology, Yokohama 226-8503, Japan*

PACS: 74.78.-w, 73.50.-h, 61.05.cp, 71.38.Mx

*Author to whom correspondence should be addressed; Electronic mail: k-yoshi@apc.titech.ac.jp

Growth of TiO and Ti_2O_3 films using pulsed laser deposition under Ar atmosphere.

In order to demonstrate the reductive effects of Ar gas, we have grown TiO and Ti_2O_3 films by using pulsed laser deposition under Ar atmosphere. Here, these films were grown at 1000°C and under Ar: 1×10^{-2} Torr and 1×10^{-3} Torr, respectively. Figure S1 shows out-of-plane XRD patterns for TiO and Ti_2O_3 films on α-Al_2O_3 (0001) substrates. The single peak was observed at $2\theta \approx 37.6°$ and 39.2°, corresponding to $d = 2.396$ Å and 2.296 Å, respectively, except for α-Al_2O_3 0006 reflections observed at $2\theta = 41.67°$. The former peak was identified as TiO 111 reflection [S1] and the latter one was identified to Ti_2O_3 0006 reflection [S2]. The XRD patterns suggest that the single-phase TiO and Ti_2O_3 films are successfully grown under Ar atmosphere by laser ablation of the target with the identical TiO_x ceramic target.

Surface morphology strongly supports the formation of TiO and Ti_2O_3 films. The insets of Fig. S1 show AFM images of the TiO and Ti_2O_3 films. As for the TiO film, the triangular facets were clearly observed, reflecting fully epitaxial growth on the hexagonal substrate as well as a rock-salt type structure exposing more stable (100) facets. The Ti_2O_3 film exhibited a trace of the spiral growth: islands surrounded by multilevel terraces originating from screw or half-loop dislocations [S3].

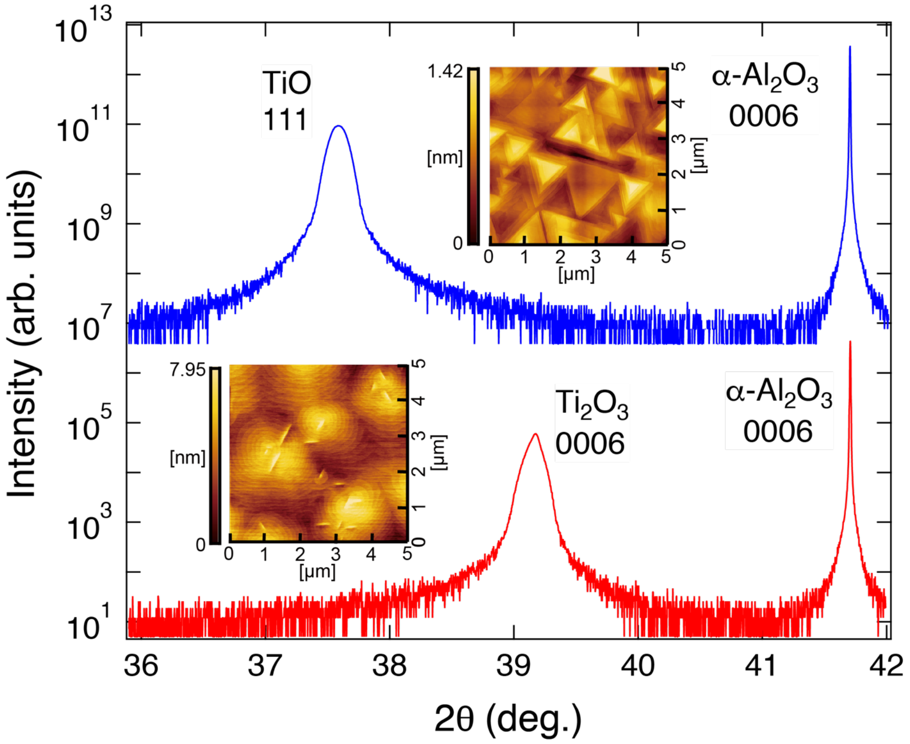

Figure S1. Out-of-plane XRD patterns for TiO and Ti_2O_3 films. The insets show atomic force microscopic images of the films.

X-ray diffraction (XRD) measurements for the Ti_4O_7 films

Figure S2 shows contour map of film- and substrate-reflections intensity, plotted against scattering angle 2θ and tilt angle χ, for the Ti_4O_7 films grown under P_{Ar} = 1 × 10^{-3} Torr (superconducting Ti_4O_7). The peak locations corresponding to the film and substrate reflections were verified. Based on this survey, we have performed synchrotron radiation XRD at BL15XU in SPring-8 for both the insulating and superconducting Ti_4O_7 films. The measured reflection profiles were shown in Figs. S3 (a–h) (insulating Ti_4O_7 film) and Figs. S4 (a–h) (superconducting Ti_4O_7 film). Signal to noise ratio was significantly improved using high-flux synchrotron radiation. From the d values of interplanar spacing distances and χ angles, the Miller indices were assigned as listed in Tables S1. For the Ti_4O_7 134 reflection, the XRD azimuth ϕ-scans around the film normal were also performed to reveal the rotational domains of the films (Figs. S5). The peaks appeared every 90°, indicating four-fold rotational domains in the films. From these XRD analyses, the in-plane (out-of-plane) epitaxial relationships between the films and substrate were determined to be Ti_4O_7 [010] // $(LaAlO_3)_{0.3}$–$(SrAl_{0.5}Ta_{0.5}O_3)_{0.7}$ (LSAT) [010], [001] and Ti_4O_7 [0–10] // LSAT [010], [001] (Ti_4O_7 [101] // LSAT [100]). Using the d values and Miller indices, we evaluated the lattice parameters of the Ti_4O_7 films in Table S2.

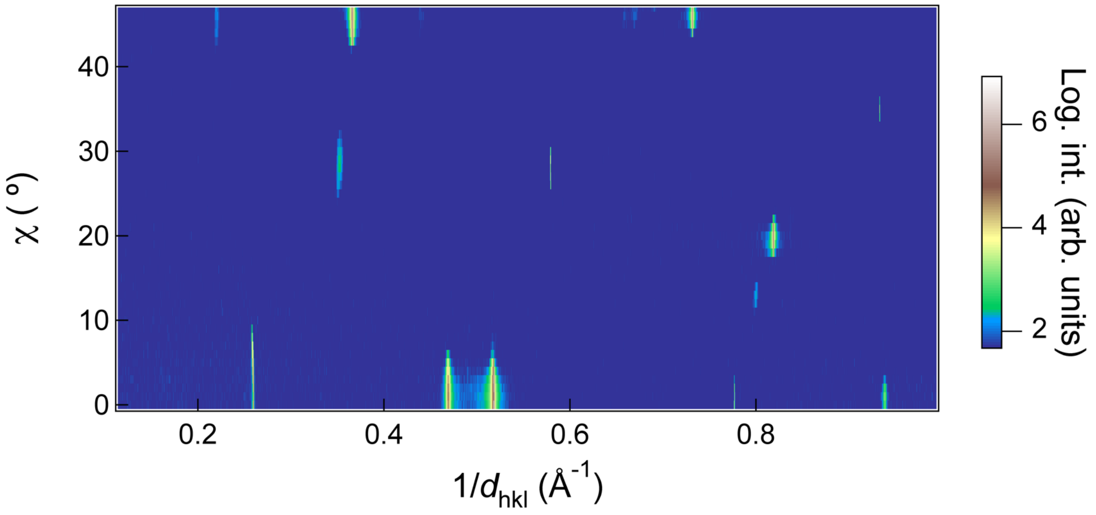

Figure S2. Contour map of film- and substrate-reflections intensity, constructed from 2θ-θ profiles measured by stepwisely varying tilt angle χ, for the superconducting Ti_4O_7 film grown on the LSAT (100) substrate.

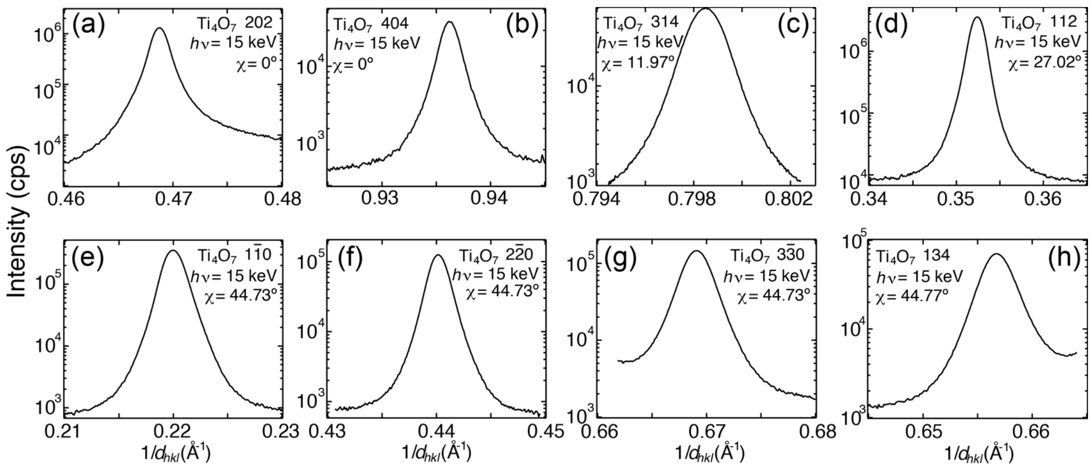

Figure S3. Some of the film-reflection profiles measured for the insulating Ti_4O_7 film. (a) 202, (b) 404, (c) 314, (d) 112, (e) 1–10, (f) 2–20, (g) 3–30, and (h) 134 reflections.

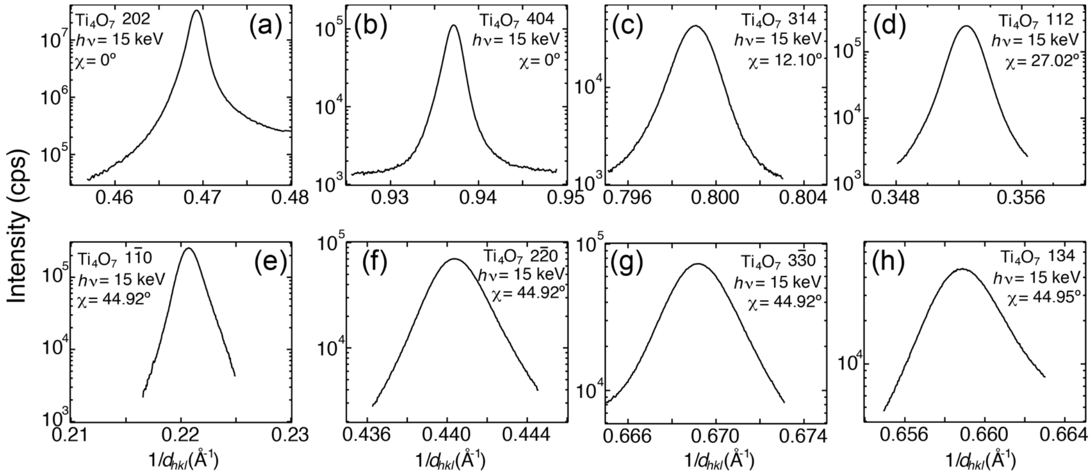

Figure S4. Some of the film-reflection profiles measured for the superconducting Ti_4O_7 film. (a) 202, (b) 404, (c) 314, (d) 112, (e) 1–10, (f) 2–20, (g) 3–30, and (h) 134 reflections.

Table S1. List of Miller indices, *d* values of interplanar spacing distances, and tilt angle χ for the insulating (left) and superconducting (light) Ti_4O_7 films.

No	*hkl*	d_{hkl} (Å)	χ (°)
**1**	202	2.133	0
**2**	404	1.068	0
**3**	314	1.252	11.97
**4**	112	2.837	27.02
**5**	1–10	4.544	44.73
**6**	2–20	2.272	44.73
**7**	3–30	1.494	44.73
**8**	134	1.522	44.77

No	*hkl*	d_{hkl} (Å)	χ (°)
**1**	202	2.131	0
**2**	404	1.067	0
**3**	314	1.251	12.10
**4**	112	2.837	27.02
**5**	1–10	4.531	44.92
**6**	2–20	2.274	44.92
**7**	3–30	1.457	44.92
**8**	134	1.518	44.95

Table S2. List of lattice parameters of insulating and superconducting Ti_4O_7 films. The lattice parameters of bulk Ti_4O_7 are also listed for comparison [1,2].

	**Insulating Ti_4O_7 film**	**Superconducting Ti_4O_7 film**	**Bulk Ti_4O_7**
a **(Å)**	5.52	5.52	5.597
b **(Å)**	7.12	7.11	7.125
c **(Å)**	20.43	20.46	20.429
α **(°)**	67.5	67.5	67.7
β **(°)**	57.3	57.2	57.16
γ **(°)**	108.8	108.8	108.76

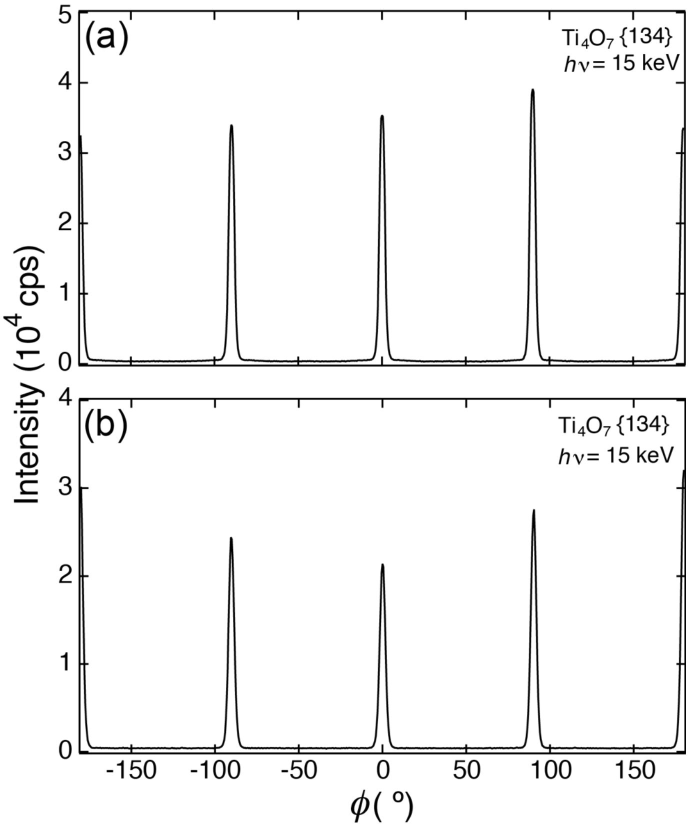

Figure S5. XRD azimuth ϕ-scans of the Ti_4O_7 134 reflections for the (a) insulating and (b) superconducting Ti_4O_7 films.

XRD measurements for the γ-Ti_3O_5 film

Figure S6 shows contour map of film- and substrate-reflections profiles intensity, plotted against scattering angle 2θ and tilt angle χ, for the γ-Ti_3O_5 film grown on α-Al_2O_3 (0001) substrates. The *d* values of interplanar spacing distances, χ angles obtained by synchrotron radiation XRD measurements (Figs. S7), and corresponding the Miller indices are listed in Table S3. For the γ-Ti_3O_5 143 reflection, the XRD azimuth ϕ-scan around the film normal was also carried out (Fig. S8). The reflections appeared every 60°, indicating the six-fold rotational domains in the film. The in-plane (out-of-plane) orientation relationships were determined to be γ-Ti_3O_5 [100] // α-Al_2O_3 [10–10], [01–10], [–1100] and γ-Ti_3O_5 [–100] // α-Al_2O_3 [10–10], [01–10], [–1100] (γ-Ti_3O_5 [011] // α-Al_2O_3 [0001]). Using the *d* values and Miller indices, we evaluated the lattice parameters of the γ-Ti_3O_5 film in Table S4.

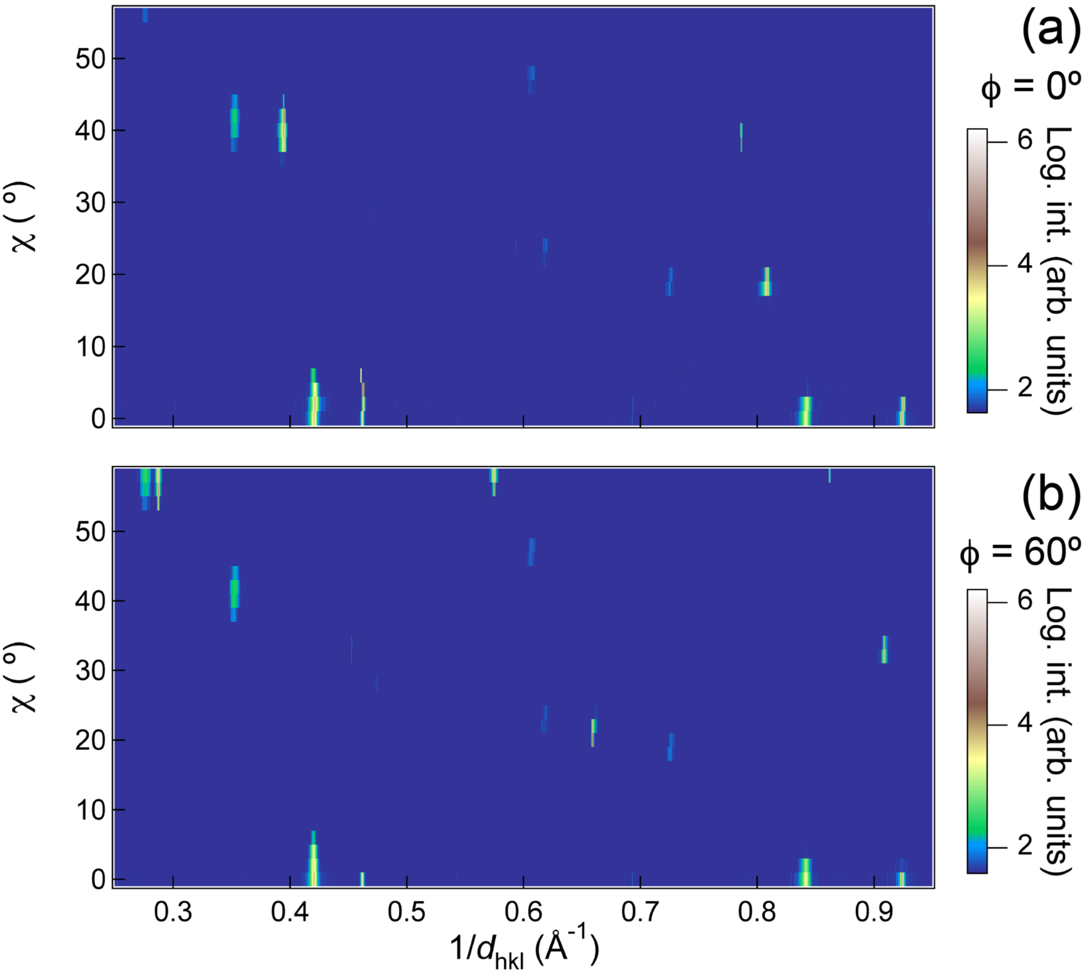

Figure S6. Contour map of film- and substrate-reflections intensity, constructed from 2θ-θ XRD measured by stepwisely varying tilt angle χ while fixing azimuth angle (a) $\phi = 0°$ and (b) $\phi = 60°$ for the γ-Ti_3O_5 film grown on α-Al_2O_3 (0001) substrates.

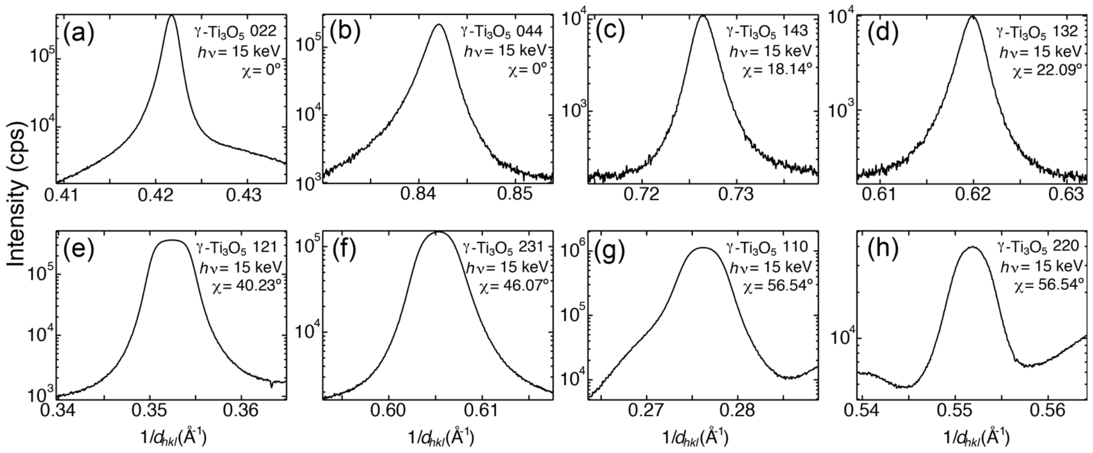

Figure S7. Some of the film-reflection profiles measured for the γ-Ti_3O_5 film. (a) 022, (b) 044, (c) 143, (d) 132, (e) 121, (f) 231, (g) 110, and (h) 220 reflections.

Table S3. List of Miller indices, d values of interplanar spacing distances, and tilt angle χ for the γ-Ti_3O_5 film.

No	*hkl*	d_{hkl} (Å)	χ (°)
**1**	022	2.375	0
**2**	044	1.188	0
**3**	143	1.377	18.14
**4**	132	1.613	22.09
**5**	121	2.839	40.23
**6**	231	1.652	46.07
**7**	110	3.620	56.54
**8**	220	1.812	56.54

Table S4. List of lattice parameters of the γ-Ti_3O_5 film. The cell parameters of bulk γ-Ti_3O_5 are also listed for comparison [4].

	γ-Ti_3O_5 film	Bulk γ-Ti_3O_5
a (Å)	4.99	5.0747
b (Å)	9.80	9.9701
c (Å)	7.06	7.1810
α (°)	110.3	109.865

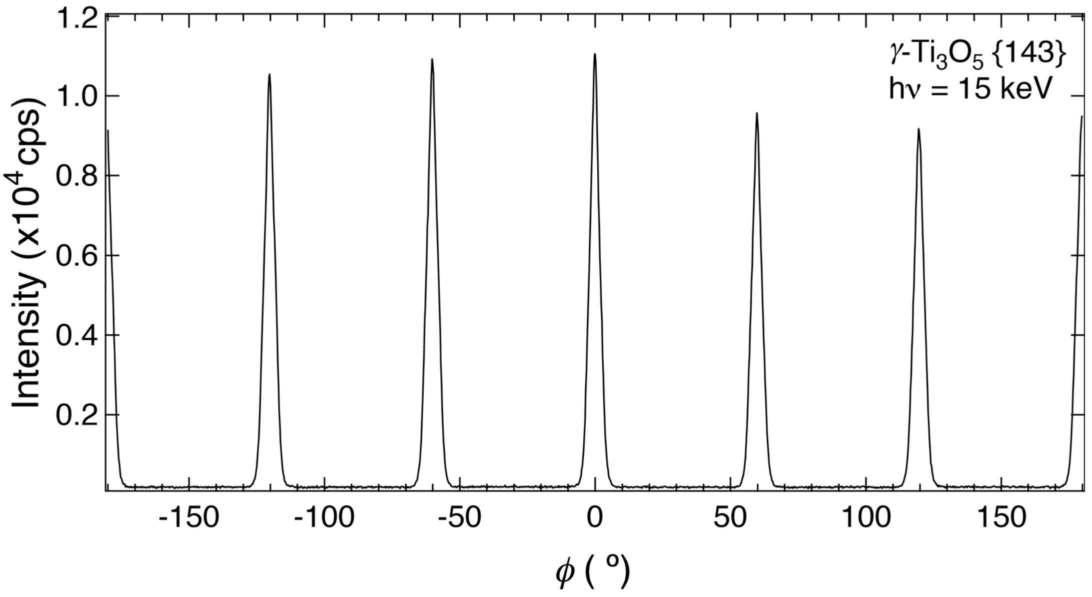

Figure S8. XRD azimuth ϕ-scans of the γ-Ti_3O_5 143 reflections.

Growth of γ-Ti_3O_5 and insulating Ti_4O_7 films in the same run

When both of α-Al_2O_3 (0001) and LSAT (100) substrates were loaded in the PLD chamber, titanate films with different structures were obtained under the condition where insulating Ti_4O_7 films were obtained on the LSAT (100) (substrate temperature of 900°C and oxygen partial pressure of 1 × 10^{-7} Torr). Figure S9(a) shows out-of-plane XRD patterns of the titanate films grown on α-Al_2O_3 (0001) and LSAT (100) substrates. The reflections coming from the titanate films were found at 2θ = 37.83 and 42.38°. The former and latter were identical to the detection angles of γ-Ti_3O_5 022 and Ti_4O_7 202 reflections, respectively. Moreover, from the temperature dependence of resistivity [Fig. S9(b)], superconductivity (metal-insulator transition) was observed for the γ-Ti_3O_5 (Ti_4O_7) films.

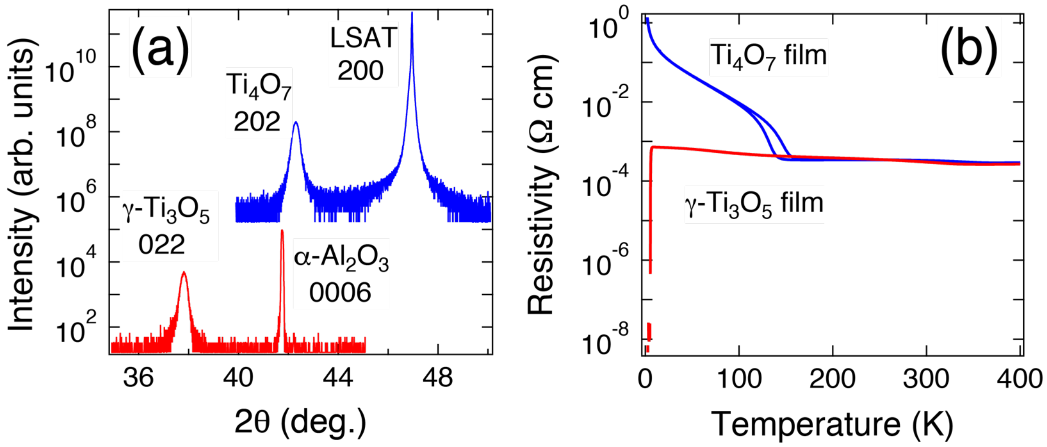

Figure S9. (a) Out-of-plane XRD patterns and (b) temperature dependence of resistivity for Ti_4O_7 and γ-Ti_3O_5 films grown in the same run.

Superconductivity in Ti_4O_7 films grown under $P_{Ar} = 1 \times 10^{-6}$ Torr

Figure S10 shows temperature dependence of resistivity for the Ti_4O_7 film grown under $P_{Ar} = 1 \times 10^{-6}$ Torr. The P_{O2} (residual oxygen gases) in the chamber is expected to be in an intermediate range between those for the growth of insulating ($P_{O2} = 1 \times 10^{-7}$ Torr) and superconducting ($P_{Ar} = 1 \times 10^{-3}$ Torr) films. Clear hysteresis was found at around 150 K [Fig. S10(a)], corresponding to the metal–insulator transition (MIT) in the normal state. In addition, the superconducting state was also found at low temperatures [Fig. S10(b)]. $T_{C,\,onset}$ of 2.9 K was slightly lower than that described in the main text. The emergence of the MIT and superconductivity in a sample supports bipolaronic mechanism in the Ti_4O_7 film.

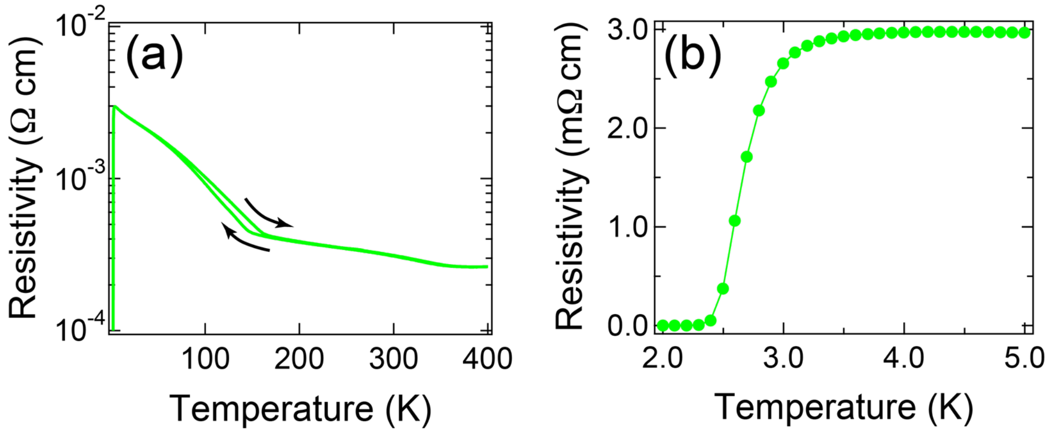

Figure S10. Temperature dependence of resistivity for the Ti_4O_7 film grown under $P_{Ar} = 1 \times 10^{-6}$ Torr (a) in the whole and (b) a low-temperatures range.

Superconductivity in Ti_4O_7 films grown on $MgAl_2O_4$ (100) substrates

Crystal structure of the Ti_4O_7 film grown on $MgAl_2O_4$ (100) substrate was investigated by XRD with Cu Kα_1 radiation. Figure S11(a) shows 2θ-θ XRD patterns of the film with various tilt angle χ. For comparison, 2θ-θ XRD patterns of the Ti_4O_7 film grown on LSAT (100) substrate were shown in Fig. S11(b). The film reflections were found at the similar angles, indicating that the film on $MgAl_2O_4$ (100) substrate was out-of-plane (101)-oriented Ti_4O_7.

Figure S12 shows temperature dependence of resistivity for the film. The resistivity curve was in good agreement with that grown under $P_{Ar} = 1 \times 10^{-3}$ Torr on LSAT (100) substrate. Superconductivity was clearly observed at low temperatures. Emergence of superconductivity of Ti_4O_7 films on the different substrates confirms that superconducting phase at low temperatures is intrinsic to the Ti_4O_7 films themselves. Furthermore, superconductors composed of Mg, Al, Ti, and O with T_c of more than 3 K are not yet known.

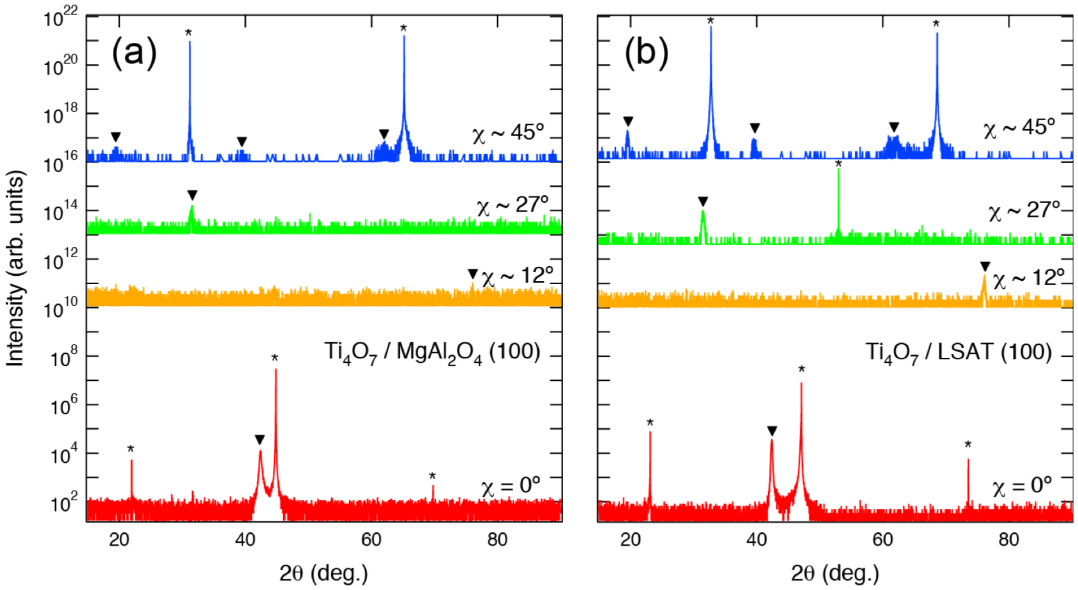

Figure S11. 2θ-ω XRD patterns of Ti_4O_7 films grown on (a) $MgAl_2O_4$ (100) and (b) LSAT (100) substrates with various tilt angle χ. The asterisks and triangles indicate the substrates and films reflections, respectively.

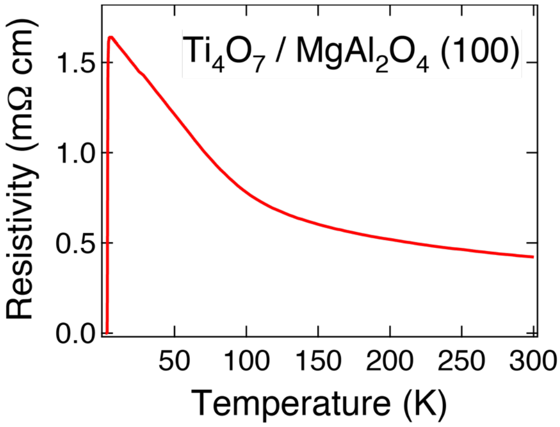

Figure S12. Temperature dependence of resistivity for Ti_4O_7 film grown on $MgAl_2O_4$ (100) substrate.

References

[S1] M. A. Afifi *et al.*, "Transport properties of polycrystalline TiO_2 and Ti_2O_3 as semiconducting oxides" J. Alloys Compd. **455**, 92 (2008).

[S2] I. Sunagawa *et al.*, "Observation and interpretation of eccentric growth spirals" J. Cryst. Growth **42**, 121 (1977).

[S3] C. Schlenker *et al.*, "Metal-insulator transitions and phase diagram of $(Ti_{1-x}V_x)_4O_7$: electrical, calorimetric, magnetic and EPR studies" J. Phys. C: Solid State Phys. **12**, 3503 (1979).
